# Infarct-related chronic total coronary occlusion and the risk of ventricular tachyarrhythmic events in out-of-hospital cardiac arrest survivors

**DOI:** 10.1007/s12471-021-01578-3

**Published:** 2021-05-27

**Authors:** M. van der Graaf, L. S. D. Jewbali, J. S. Lemkes, E. M. Spoormans, M. van der Ent, M. Meuwissen, M. J. Blans, P. van der Harst, J. P. Henriques, A. Beishuizen, C. Camaro, G. B. Bleeker, N. van Royen, S. C. Yap

**Affiliations:** 1 Department of Cardiology, Erasmus MC, University Medical Centre Rotterdam, Rotterdam, The Netherlands; 2 Department of Cardiology, Amsterdam University Medical Centre VUMC, Amsterdam, The Netherlands; 3 Department of Cardiology, Maasstad Hospital, Rotterdam, The Netherlands; 4 Department of Cardiology, Amphia Hospital, Breda, The Netherlands; 5 Department of Intensive Care Medicine, Rijnstate Hospital, Arnhem, The Netherlands; 6 Department of Cardiology, University Medical Centre Groningen, Groningen, The Netherlands; 7 Department of Cardiology, Heart Center, Amsterdam UMC, University of Amsterdam, Amsterdam Cardiovascular Sciences, Amsterdam, The Netherlands; 8 Department of Intensive Care, Medisch Spectrum Twente, Enschede, The Netherlands; 9 Department of Cardiology, Radboud University Medical Centre, Nijmegen, The Netherlands; 10 Department of Cardiology, Haga Hospital, The Hague, The Netherlands

**Keywords:** Chronic total occlusion, Ventricular tachycardia, Out-of-hospital cardiac arrest, Implantable cardioverter-defibrillator

## Abstract

**Introduction:**

Chronic total coronary occlusion (CTO) has been identified as a risk factor for ventricular arrhythmias, especially a CTO in an infarct-related artery (IRA). This study aimed to evaluate the effect of an IRA-CTO on the occurrence of ventricular tachyarrhythmic events (VTEs) in out-of-hospital cardiac arrest survivors without ST-segment elevation.

**Methods:**

We conducted a post hoc analysis of the COACT trial, a multicentre randomised controlled trial. Patients were included when they survived index hospitalisation after cardiac arrest and demonstrated coronary artery disease on coronary angiography. The primary endpoint was the occurrence of a VTE, defined as appropriate implantable cardioverter-defibrillator (ICD) therapy, sustained ventricular tachyarrhythmia or sudden cardiac death.

**Results:**

A total of 163 patients from ten centres were included. Unrevascularised IRA-CTO in a main vessel was present in 43 patients (26%). Overall, 61% of the study population received an ICD for secondary prevention. During a follow-up of 1 year, 12 patients (7.4%) experienced at least one VTE. The cumulative incidence rate of VTEs was higher in patients with an IRA-CTO compared to patients without an IRA-CTO (17.4% vs 5.6%, log-rank *p* = 0.03). However, multivariable analysis only identified left ventricular ejection fraction < 35% as an independent factor associated with VTEs (adjusted hazard ratio 8.7, 95% confidence interval 2.2–35.4). A subanalysis focusing on CTO, with or without an infarct in the CTO territory, did not change the results.

**Conclusion:**

In out-of-hospital cardiac arrest survivors with coronary artery disease without ST-segment elevation, an IRA-CTO was not an independent factor associated with VTEs in the 1st year after the index event.

**Supplementary Information:**

The online version of this article (10.1007/s12471-021-01578-3) contains supplementary material, which is available to authorized users.

## What’s new?

- Previous implantable cardioverter-defibrillator studies have demonstrated a relationship between the presence of a chronic total coronary occlusion (CTO), especially in an infarct-related artery (IRA), and ventricular tachyarrhythmic events (VTEs).
- An IRA-CTO is a common phenomenon in out-of-hospital cardiac arrest survivors, but was not an independent factor for VTEs in the 1st year after the index event.
- The only independent factor associated with VTEs in the 1st year was the presence of severe left ventricular dysfunction.

## Introduction

Chronic total coronary occlusion (CTO) is common (approximately 20%) in patients presenting for diagnostic coronary angiography [1, 2]. A CTO is associated with chronic hibernating myocardium in the CTO territory, which can lead to electrical instability and may increase the risk of ventricular arrhythmias [3–5]. The exact role of a CTO in the pathophysiological mechanism of ventricular arrhythmias is not fully understood. Several studies in patients who received an implantable cardioverter-defibrillator (ICD) for primary or secondary prevention demonstrated a higher risk of appropriate ICD therapy and all-cause mortality in patients with a CTO [6–11]. A recent single-centre study demonstrated that the presence of a CTO is an independent predictor of appropriate ICD therapy in out-of-hospital cardiac arrest (OHCA) survivors with coronary artery disease who received an ICD for secondary prevention [11]. However, studies have suggested that a CTO in an infarct-related artery (IRA) is a better predictor of ventricular arrhythmias than a CTO in a non-IRA [9, 10]. The aim of the current study is to investigate the impact of an IRA-CTO on the incidence of ventricular arrhythmias in OHCA survivors with coronary artery disease who present without ST-segment elevation.

## Methods

### Study population

The present study is a post hoc analysis of the COACT (Coronary Angiography after Cardiac Arrest) trial, a multicentre randomised controlled trial that was conducted in 19 Dutch hospitals [12, 13]. Patients were included in the period between January 2015 and July 2018. Overall, 552 OHCA patients without ST-segment elevation were included, who were randomly assigned to undergo immediate coronary angiography or coronary angiography after neurological recovery. The COACT trial demonstrated that a strategy of immediate angiography was not superior to a strategy of delayed angiography with respect to survival at 90 days [13]. The 11 centres with the highest enrolment rates were approached, of which 10 centres participated in this study. Our study population included patients who survived their index hospitalisation and displayed coronary artery disease on their coronary angiogram.

### Study endpoints

In this study, a CTO was defined as a total coronary occlusion in a major epicardial coronary artery with Thrombolysis in Myocardial Infarction (TIMI) 0 flow and an estimated occlusion duration of ≥ 3 months [9]. Previously chronic occluded vessels that were surgically or percutaneous revascularised were not defined as CTO in this study. An IRA-CTO was defined as a CTO associated with a previous myocardial infarction in the territory of the coronary artery. Previous myocardial infarction had to be documented by Q waves on electrocardiography (ECG) and/or evidence of scar on imaging, such as regional wall motion abnormalities on echocardiography or late gadolinium enhancement on cardiac magnetic resonance imaging.

Patients were followed during 1 year after inclusion or until date of death, whichever occurred first. The primary endpoint was defined as the occurrence of a ventricular tachyarrhythmic event (VTE) after hospital discharge. A VTE was defined as appropriate ICD therapy (antitachycardia pacing and/or shock for ventricular tachyarrhythmia), sudden cardiac death (SCD) presumably due to ventricular tachyarrhythmia or documented sustained ventricular tachycardia or ventricular fibrillation during follow-up. Secondary endpoints were all-cause mortality and cardiac mortality.

### Statistical analysis

Continuous variables are presented as mean ± standard deviation or median (interquartile range). Categorical variables are presented as frequencies with percentages. Continuous variables were compared between groups using the Student *t*-test or the Mann-Whitney U test. Categorical variables were compared using the chi-squared test or the Fisher exact test. The cumulative incidence rate for VTEs was calculated and compared between groups using Kaplan-Meier survival analysis. Time zero was the date of the cardiac arrest. To evaluate whether an IRA-CTO is independently associated with the occurrence of a VTE, multivariable Cox regression analysis was used. IRA-CTO and any variable with a *p*-value < 0.10 in the univariable analysis were entered in a multivariable regression model. Unless otherwise specified, a significance level of α = 0.05 was used. Statistical analysis was performed using SPSS version 25.0 (IBM Corporation, Armonk, NY, USA).

## Results

**Fig. 1:**
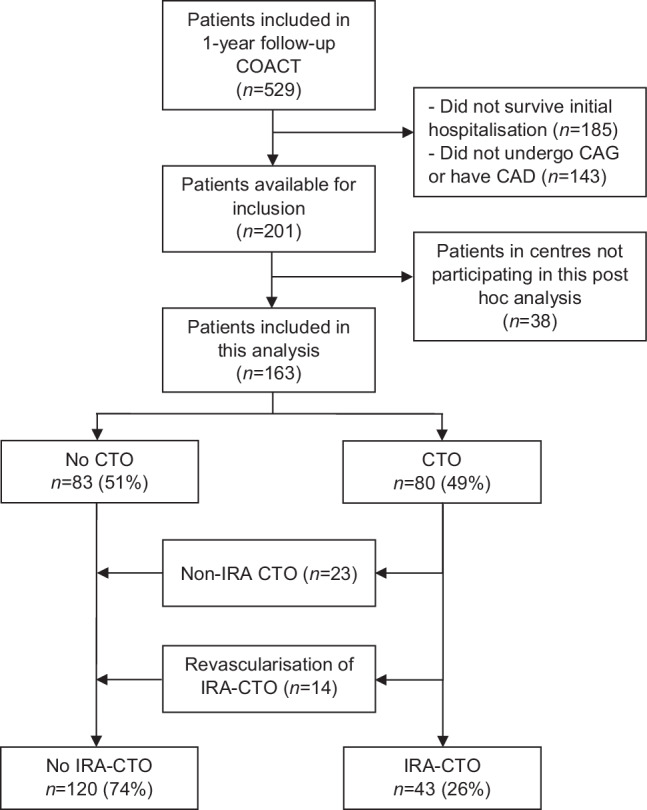
Flow chart of study population. *CAD* coronary artery disease, *CAG* coronary angiography, *COACT* Coronary Angiography after Cardiac Arrest trial, *CTO* chronic total coronary occlusion, *IRA-CTO* infarct-related artery chronic total coronary occlusion

A total of 163 patients with coronary artery disease from ten centres were included, who survived the index hospitalisation (Fig. 1). Of the 80 patients (49%) with a CTO in a main vessel, 57 patients had an IRA-CTO based on Q waves on ECG and/or cardiac imaging studies. Of these patients, 14 patients underwent successful percutaneous or surgical revascularisation of an IRA-CTO, leading to 43 patients (26%) with at least one remaining IRA-CTO (Fig. 1). Baseline patient characteristics are displayed in Tab. 1. Patients in the IRA-CTO group more often had a history of a previous myocardial infarction, more frequently had a left ventricular ejection fraction (LVEF) < 35% and multivessel disease compared to patients without an IRA-CTO. Patients without an IRA-CTO were more likely to undergo a percutaneous coronary intervention (PCI), while patients with an IRA-CTO more often received pharmacological or conservative treatment (*p* < 0.01) during the index hospitalisation. Overall, 61% of the study population received an ICD for secondary prevention. Patients with an IRA-CTO were more likely to receive an ICD.

**Table 1:** Baseline characteristics

Characteristic	All patients (*n* = 163)	No IRA-CTO group (*n* = 120)	IRA-CTO group (*n* = 43)	*p*-value
Age, years	64 ± 10	64 ± 11	66 ± 9	0.21
Sex, male	148 (91)	108 (90)	40 (93)	0.76
*Medical history*				
– Diabetes mellitus	23 (14)	20 (17)	3 (7)	0.13
– Hypertension	80 (49)	60 (50)	20 (48)	0.86
– Previous MI	50 (31)	27 (23)	23 (54)	< 0.01
– Previous PCI	33 (20)	22 (18)	11 (26)	0.38
– Previous CABG	14 (9)	7 (6)	7 (16)	0.05
– Hypercholesterolaemia	53 (33)	37 (31)	16 (39)	0.34
– Renal dysfunction (MDRD-GFR < 60)	22 (14)	15 (13)	7 (16)	0.60
– LVEF ≤ 35% (*n* = 159)	40 (25)	22 (19)	18 (43)	< 0.01
– Multivessel disease	91 (56)	59 (49)	33 (74)	< 0.01
*Treatment after CAG*				< 0.01
– Conservative	37 (23)	15 (13)	22 (51)	
– CABG	26 (16)	21 (18)	5 (12)	
– PCI during first CAG	68 (42)	57 (48)	11 (26)	
– Staged PCI	27 (17)	22 (18)	5 (12)	
– PCI during first CAG – and staged	5 (3)	5 (4)	0 (0)	
*Medication at discharge (n* *=* *160)*				
– β-blocker	151 (94)	110 (94)	41 (95)	1.00
– ACE inhibitor	133 (83)	98 (84)	35 (81)	0.81
– Statin	143 (89)	102 (87)	41 (95)	0.16
– Diuretics	59 (37)	40 (34)	19 (44)	0.27
– Amiodarone	10 (6)	7 (6)	3 (7)	0.73
– Digoxin	7 (4)	6 (5)	1 (2)	0.68
ICD implantation	99 (61)	62 (52)	37 (86)	< 0.01

Results are presented as mean ± SD or count (percentage)

*CABG* coronary artery bypass graft, *CAG* coronary angiography, *GFR* glomerular filtration rate, *ICD* implantable cardioverter-defibrillator, *IRA-CTO* infarct-related artery chronic total coronary occlusion, *LVEF* left ventricular ejection fraction, *MDRD* Modification of Diet in Renal Disease, *MI* myocardial infarction, *OHCA* out-of-hospital cardiac arrest, *PCI* percutaneous coronary intervention

**Fig. 2:**
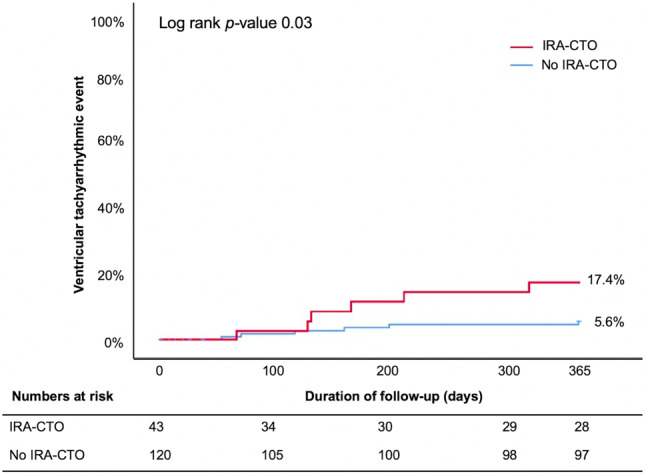
Cumulative incidence rate for ventricular tachyarrhythmic events stratified by presence or absence of infarct-related artery chronic total coronary occlusion (*IRA-CTO*)

During a follow-up of 1 year, 12 patients (7.4%) experienced at least one VTE. The VTE consisted of appropriate ICD therapy in all cases. The cumulative 1‑year event rate was 17.4% versus 5.6% in the IRA-CTO and no IRA-CTO group, respectively (log rank *p* = 0.03) (Fig. 2). Multivariable Cox regression analysis demonstrated that only LVEF < 35% was an independent factor associated with a VTE (adjusted hazard ratio (HR) 8.7, 95% confidence interval (CI) 2.2–35.4) (Tab. 2). There was no difference in the cycle length of the documented ventricular arrhythmia between patients with and without IRA-CTO (212 ± 28 ms vs 261 ± 69 ms, respectively, *p* = 0.18). Furthermore, when excluding the 14 patients with successful revascularisation from the no IRA-CTO group, the results of the multivariable analysis did not change (data not presented). These 14 patients had no VTE during follow-up.

**Table 2:** Multivariable Cox regression analysis for ventricular tachyarrhythmic events focusing on infarct-related artery chronic total coronary occlusion (*IRA-CTO*)

	Univariable Cox regression analysis		Multivariable Cox regression analysis	
	HR (95% CI)	*p*-value	HR (95% CI)	*p*-value
IRA-CTO^a^	3.2 (1.0–9.9)	0.04	1.2 (0.4–4.3)	0.74
LVEF < 35%	9.4 (2.5–35.4)	< 0.01	8.7 (2.2–35.4)	< 0.01
Multivessel disease	2.5 (0.7–9.1)	0.18		
Presence of ICD	1.0 (0.9–1.1)	0.91		

*CI* confidence interval, *HR* hazard ratio, *ICD* implantable cardioverter-defibrillator, *LVEF* left ventricular ejection fraction

^a^In comparison to patients without IRA-CTO (i.e. patients with no CTO and patients with a CTO in a non-infarct-related artery)

As a subanalysis we evaluated whether the presence of an unrevascularised CTO (*n* = 54), irrespective of a localisation in an IRA, was associated with a VTE. The cumulative 1‑year event rate was 16.1% versus 5.1% in the CTO and no CTO group, respectively (log rank *p* = 0.03). Multivariable analysis demonstrated that a CTO was also not an independent factor associated with VTEs (Electronic Supplementary Material, Table S1).

During follow-up 3 patients (1.8%) died after hospital discharge. One death was classified as a cardiac death and the other 2 deaths were classified as non-cardiac death. No SCD occurred during follow-up.

## Discussion

In OHCA survivors with coronary artery disease without ST-segment elevation at presentation, the presence of an IRA-CTO was not independently associated with a VTE within the 1st year. Only severe left ventricular (LV) dysfunction was independently associated with a higher risk for VTEs.

### Relationship between IRA-CTO and VTE

The presence of a CTO is common in patients with coronary artery disease. In the overall COACT population, thus survivors of OHCA without ST-segment elevation, a CTO was present in 36% in patients who underwent CAG [13]. In the present study, including only patients with coronary artery disease, 49% of patients had a CTO in at least one main vessel. This prevalence is comparable to previously reported prevalence rates of CTO in patients with coronary artery disease [6, 11, 13, 14]. In 71% of patients with a CTO, at least one CTO could be identified as an IRA-CTO based on ECG or cardiac imaging. While there are limited data concerning the prevalence of IRA-CTOs, this percentage is consistent with previous studies in which 56–74% of CTOs were located in an IRA [10, 15]. Several ICD studies have shown that the presence of CTO is an independent predictor for ventricular arrhythmias [6–11]. However, the study by Raja et al. did not demonstrate this relationship [16]. Recent studies showed that this discrepancy may be explained by the presence of a CTO in an IRA or a non-IRA [9, 10, 17]. There are different pathophysiological mechanisms for the increased risk of VTEs in patients with an IRA-CTO, but the most prevailing theory is that the presence of scar with areas of activation delay is a prerequisite for the induction and maintenance of reentry tachycardias [18, 19]. Furthermore, the myocardium supplied by the CTO is a chronically hibernating myocardium which is associated with proarrhythmic properties despite the presence of a well-developed collateral system [20]. The present study demonstrates that patients with an IRA-CTO have an increased risk of a VTE in the 1st year after the index event. This increased VTE risk seems to be related to a higher proportion of patients with severe LV dysfunction in the IRA-CTO group (i.e. collinearity). Severe LV dysfunction was the only independent factor associated with a higher VTE risk. LV dysfunction is a well-known risk factor for recurrent ventricular arrhythmias in survivors of cardiac arrest [6, 11]. The discrepancy in the prognostic role of a CTO between our study and previous studies may be explained by the relative short follow-up period, smaller sample size and lower proportion of ICD carriers in our study population [6, 10, 11]. Despite these limitations of our study, we can conclude that severe LV dysfunction is a stronger predictor of VTE than the presence of an IRA-CTO.

### Role of PCI for CTO

Observational studies have shown that CTO PCI may be associated with LV reverse remodelling and improvement of various electrocardiographic parameters (e.g. QT dispersion, late potentials) that are associated with ventricular arrhythmias and SCD [4, 21]. Randomised trials, however, only demonstrated improvement in regional LV function and not in global LV function [22, 23]. The number of patients who underwent a CTO PCI in our study population was too small to perform solid statistical analysis. Thus, it is not known whether CTO PCI in the specific subset of OHCA survivors without ST-segment elevation is beneficial.

### Prophylactic ICD in CTO patients

Finally, the current guidelines recommend not to implant an ICD in OHCA survivors when there is a reversible factor, such as cardiac ischaemia [24]. The majority of our study population underwent revascularisation and only 61% received an ICD. Small increases in troponin levels may present a challenge for clinicians, as it is difficult to determine whether this elevation is due to ventricular tachyarrhythmia and resuscitation or due to ischaemia causing the ventricular tachyarrhythmia. In the first case, an ICD is warranted; in the second case revascularisation seems to be sufficient. Data from the current study are reassuring, as no patient without an ICD died suddenly in the 1st year.

### Study limitations

Several limitations of this study should be considered. First, the small sample size, limited number of events and the short follow-up period hamper the power of our study to detect a potentially clinically significant relationship between IRA-CTOs and the occurrence of VTEs. Second, only 61% of the study population received an ICD, which may lead to underestimation of the true incidence of VTEs. However, patients without an ICD were deemed to be at low risk for a VTE by the treating physician. On the other hand, our study population is unique, as prior studies investigating IRA-CTOs as a potential risk factor for VTEs were limited to an ICD population [6–11].

## Conclusions

In OHCA survivors with coronary artery disease without ST-segment elevation, severe LV dysfunction, and not an IRA-CTO, was an independent factor associated with VTEs within the 1st year.

## Supplementary Information

**Table S1** Multivariable Cox regression analysis for ventricular tachyarrhythmic events focusing on chronic total coronary occlusion (*CTO*)
