# Supplementary material for: Infarct-related chronic total coronary occlusion and the risk of ventricular tachyarrhythmic events in out-of-hospital cardiac arrest survivors
Source: Neth Heart J. 2021 May 27;29(10):500–5. doi: 10.1007/s12471-021-01578-3 (PMC8455757; doi:10.1007/s12471-021-01578-3)
Supplement: Supplementary file 1 — Table S1 Multivariable Cox regression analysis for ventricular tachyarrhythmic events focusing on chronic total coronary occlusion (CTO) [file 12471_2021_1578_MOESM1_ESM.docx]

**Table S1** Multivariable Cox regression analysis for ventricular tachyarrhythmic events focusing on chronic total coronary occlusion (*CTO*)

|  | **Univariable Cox regression analysis** | | **Multivariable Cox regression analysis** | |
| --- | --- | --- | --- | --- |
|  | **HR (95% CI)** | ***p*-value** | **HR (95% CI)** | ***p*-value** |
| CTO^a^ | 3.2 (1.0-10.2) | 0.04 | 1.4 (0.4-4.9) | 0.58 |
| LVEF <35% | 9.4 (2.5-35.4) | <0.01 | 8.4 (2.1-33.7) | <0.01 |
| Multivessel disease | 2.5 (0.7-9.1) | 0.18 |  |  |
| Presence of ICD | 1.0 (0.9-1.1) | 0.91 |  |  |

*CI* confidence interval, *HR* hazard ratio, *ICD* implantable cardioverter-defibrillator, *LVEF* left ventricular ejection fraction

^a^ In comparison to patients without CTO
